# Supplementary material for: Genomic Bootstrap Barcodes and Their Application to Study the Evolution of Sarbecoviruses
Source: Viruses. 2022 Feb 21;14(2):440. doi: 10.3390/v14020440 (PMC8879460; doi:10.3390/v14020440)
Supplement: Supplementary file 1 [file viruses-14-00440-s001.zip › viruses-1566197-supplementary.pdf]

Figure S1: SuperTRI bootstrap 50%-majority-rule consensus (SB) trees reconstructed from bootstrap log files generated from the four SWB analyses based on different window sizes (250, 500, 1000, and 2000 nt).

The values at the nodes are mean bootstrap percentages (MBP) calculated under SuperTRI v57 (Ropiquet et al., 2009). All SuperTRI bootstrap percentages (SBP) calculated with the MRP matrix under PAUP* (Swofford, 2003) were equal to 100% (not shown), except for nodes for which the SBP value is indicated at the left of the slash.

A. SuperTRI bootstrap 50%-majority-rule consensus (SB) trees reconstructed from bootstrap log files generated from the SWB analysis based on a window of 250 nucleotides.

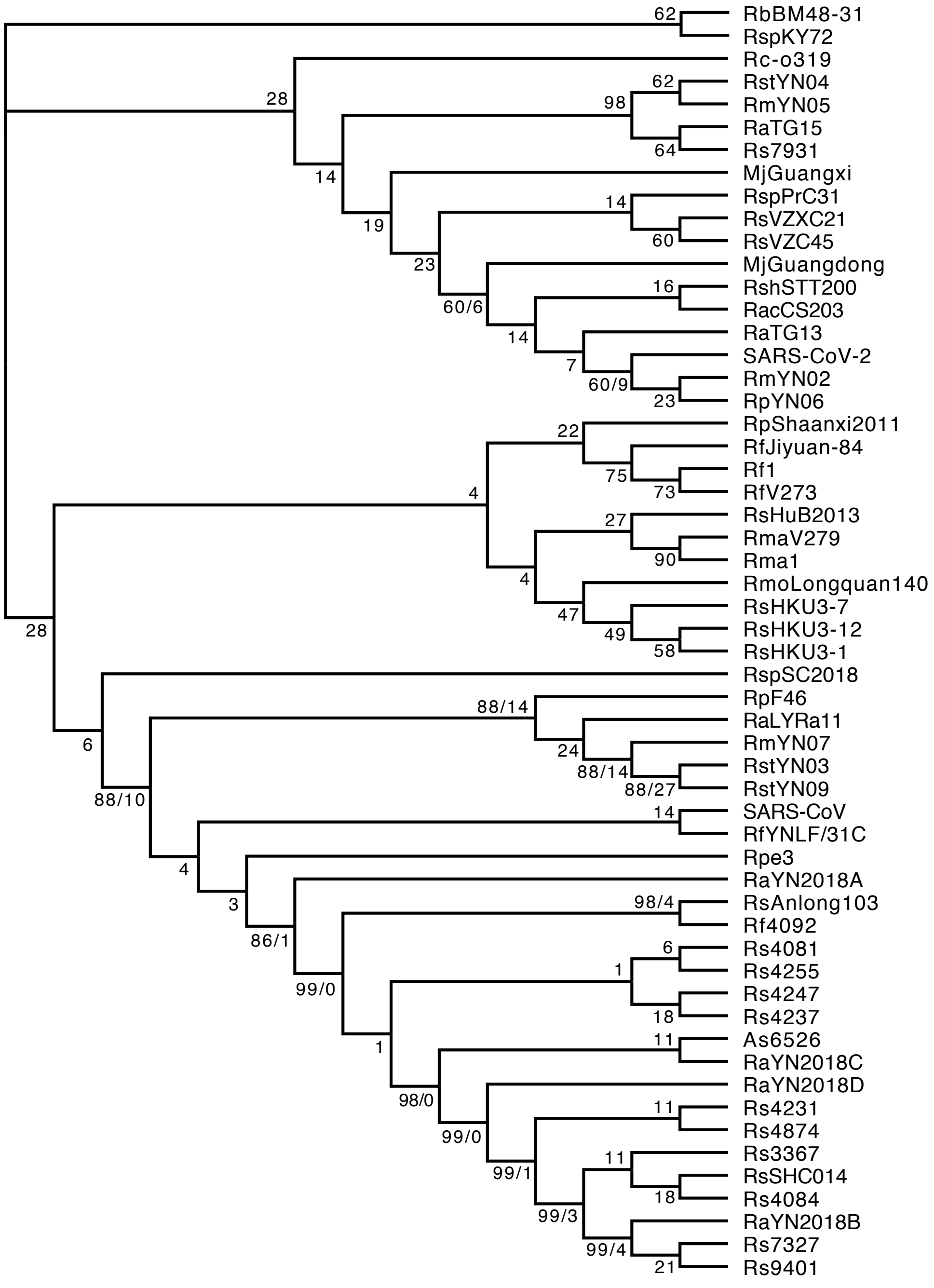

B. SuperTRI bootstrap 50%-majority-rule consensus (SB) trees reconstructed from bootstrap log files generated from the SWB analysis based on a window of 500 nucleotides.

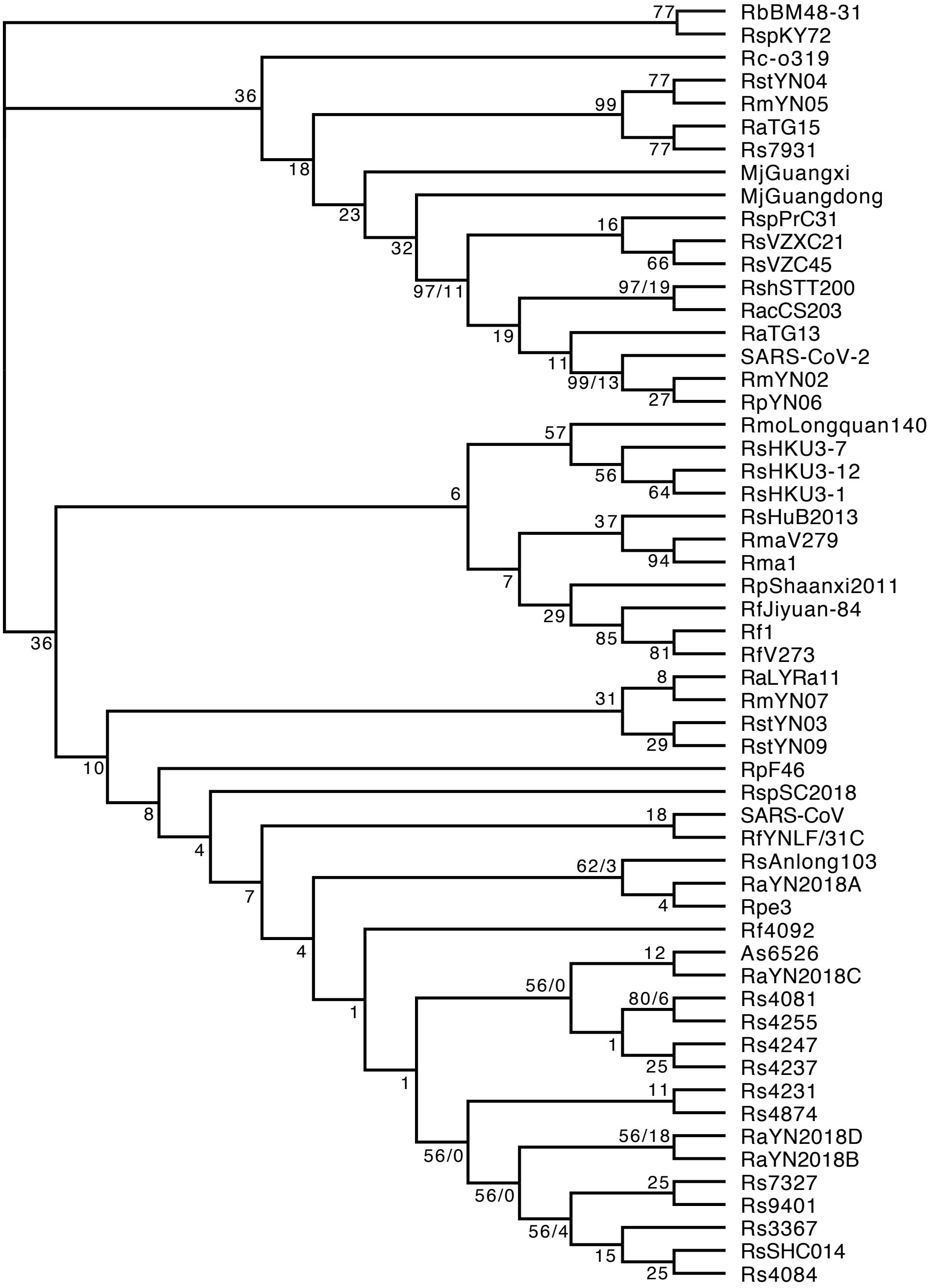

C. SuperTRI bootstrap 50%-majority-rule consensus (SB) trees reconstructed from bootstrap log files generated from the SWB analyses based on the sliding window of 1000 nucleotides.

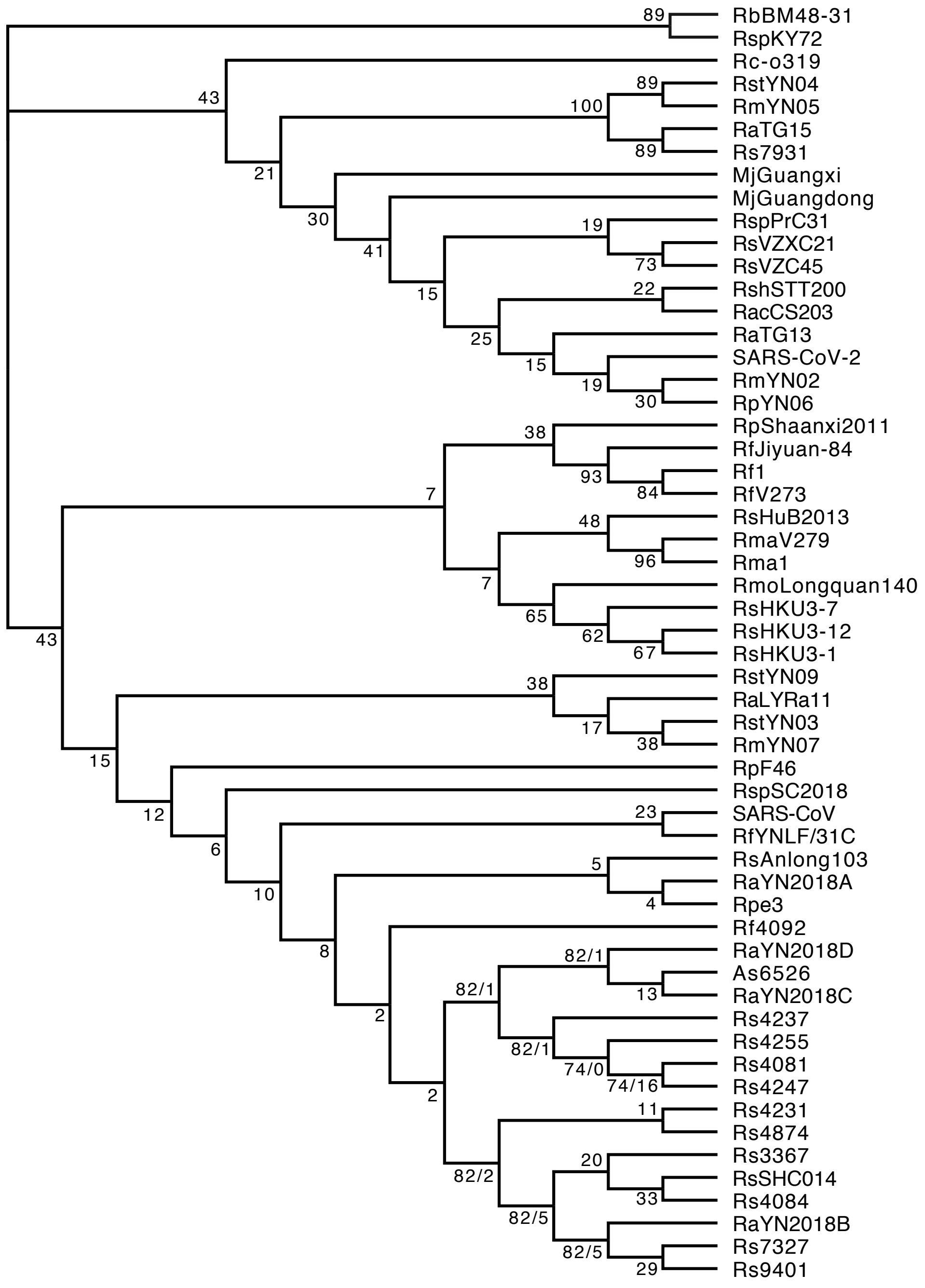

D. SuperTRI bootstrap 50%-majority-rule consensus (SB) trees reconstructed from bootstrap log files generated from the SWB analysis based a window of 2000 nucleotides.

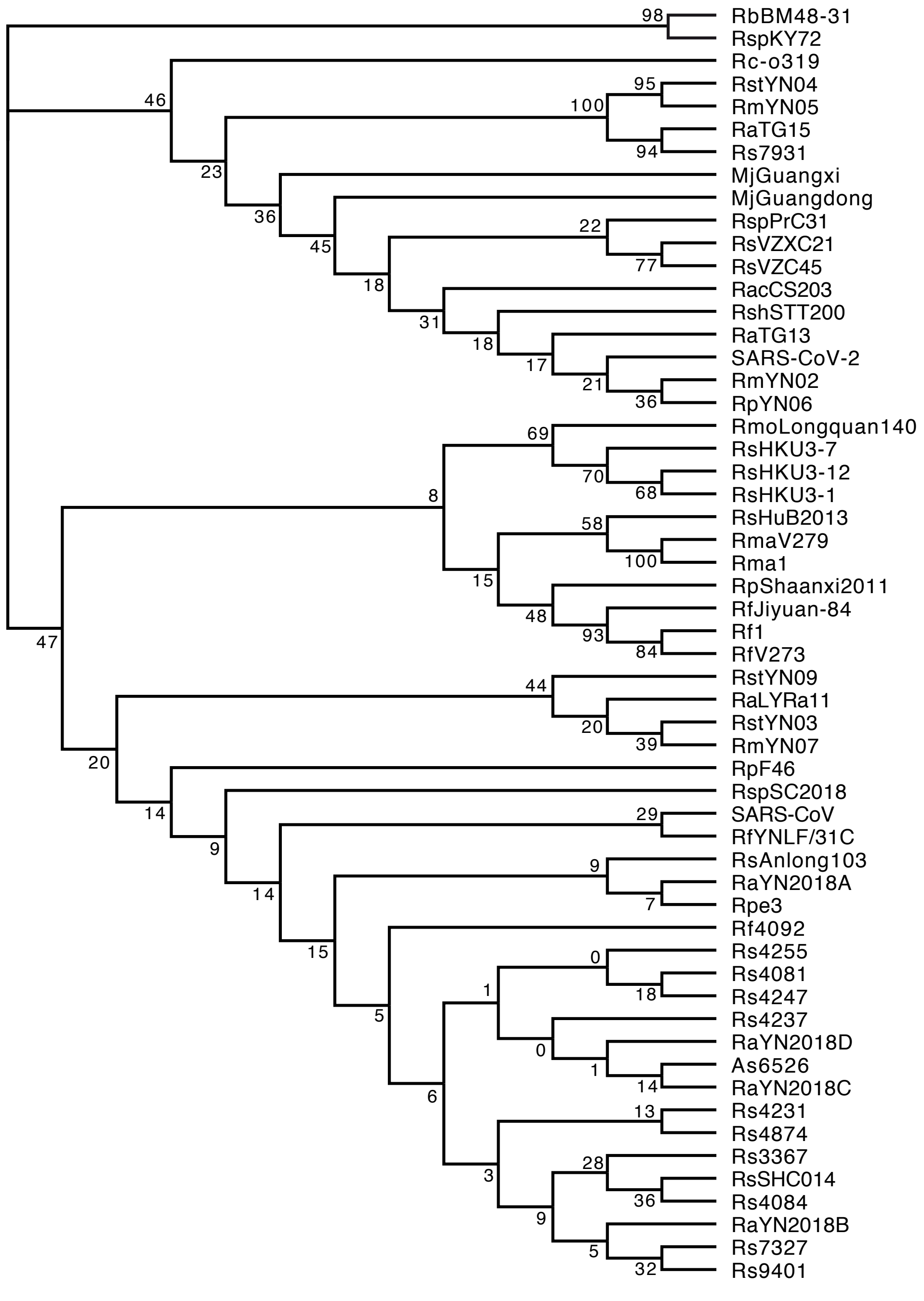

Figure S2: **Comparison between Mean Bootstrap Percentage (MBP) and reproducibility index (Rep) for all nodes of the tree of Figure 3.** The linear regression is reported in red.

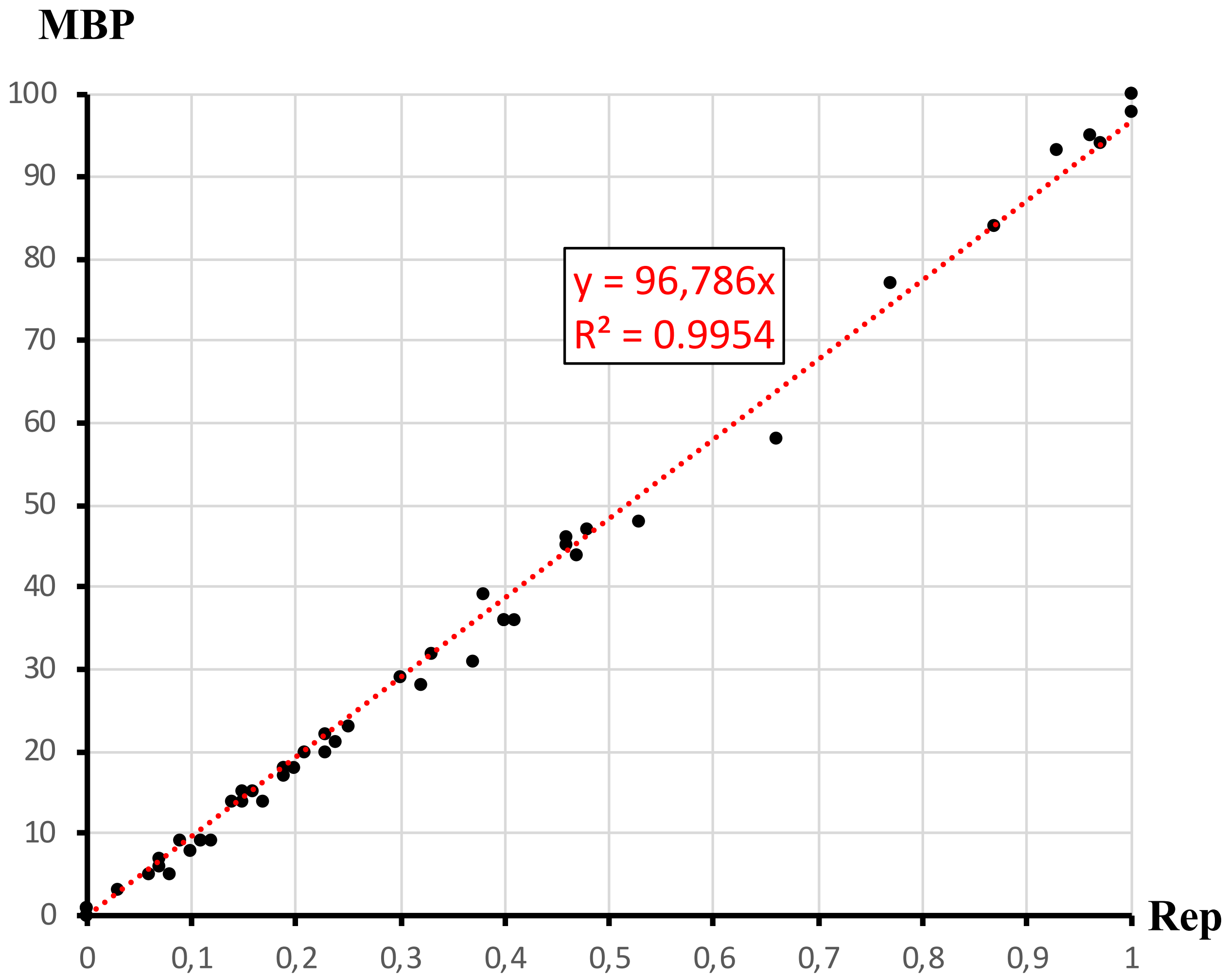

Figure S3: Synthesis tree showing the most reliable relationships reconstructed from SWB analyses of a reduced alignment of 49 *Sarbecovirus* genomes.

Bootstrap analyses were conducted on an alignment of 49 *Sarbecovirus* genomes (28,845 nt) using the SWB program and four different window sizes (250, 500, 1000 or 2000 nt).
The tree shown here is a 75% majoritary-rule consensus tree of the four SB trees reconstructed using four different window sizes. The nodes were recovered in all the four SB trees, except those indicated by dash branches, which were found monophyletic in only three of the four SB analyses. The GB barcodes were constructed for all nodes showing at least one Bootstrap Percentage (BP) ≥ 70% in the four SWB analyses using different window sizes. Thick branches highlight the nodes that were supported by Mean Bootstrap Percentage (MBP) ≥ 33% with a window size of 2000 nt.
The colours of sarbecoviruses indicate to which group of synonymous nucleotide composition they belong (Hassanin, in review): black for SARS-CoV related coronaviruses (*SCoVrC*); green for coronaviruses related to SARS-CoV-2 (*SCoV2rC*); light blue for the two pangolin viruses (*PangSar*); dark blue for the three bat *RecSar* viruses showing evidence of genomic recombination between *SCoV2rC* and *SCoVrC*; red for the four divergent bat viruses from Yunnan (*YunSar*); and orange for the bat virus from Japan.

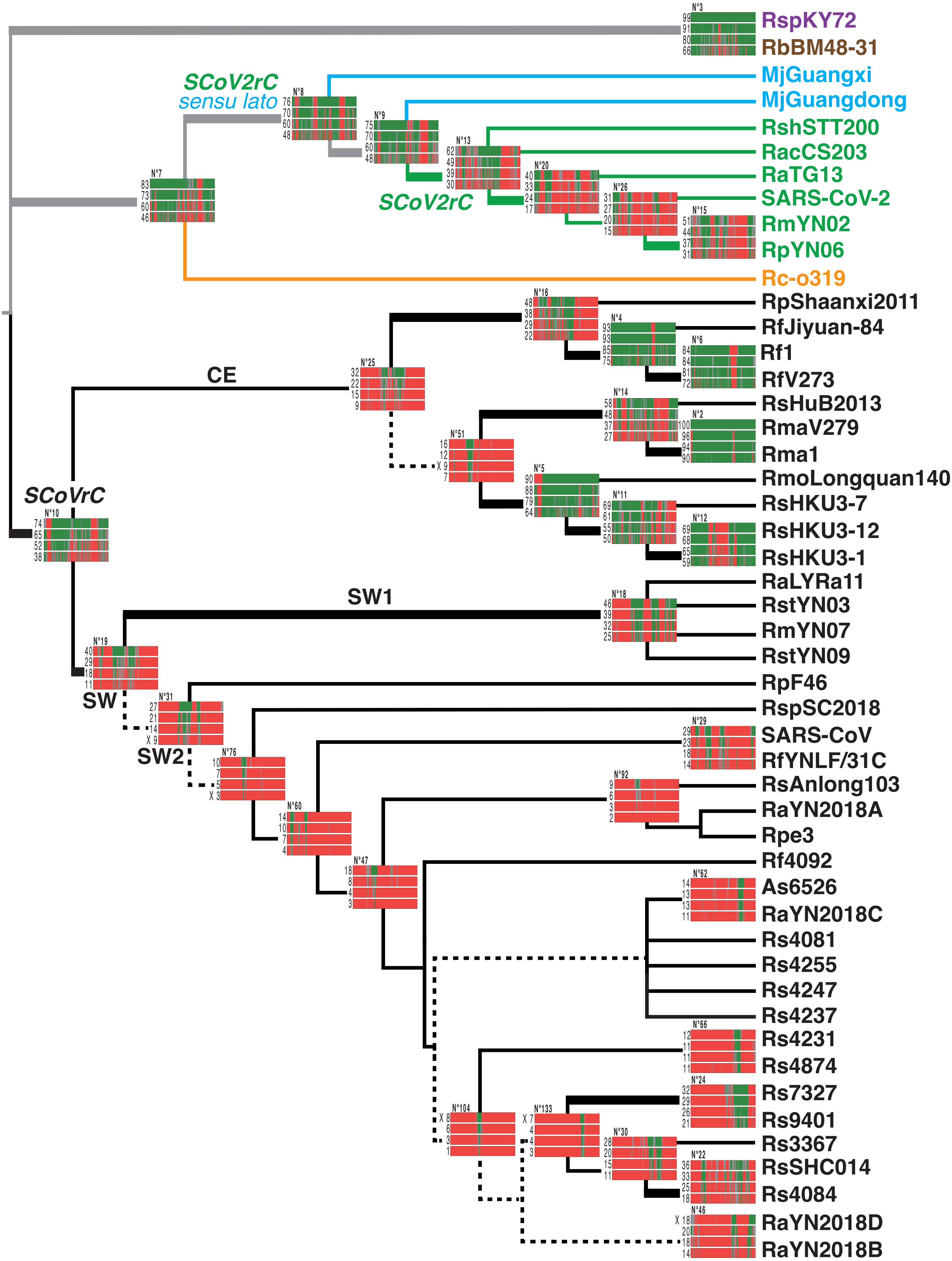

RspKY72
RbBM48-31
SCoV2rC
sensu lato
MjGuangxi
MjGuangdong
RshSTT200
RacCS203
RaTG13
SCoV2rC
SARS-CoV-2
RmYN02
RpYN06
Rc-o319
RpShaanxi2011
RfJiyuan-84
Rf1
CE
RfV273
RsHuB2013
RmaV279
Rma1
RmoLongquan140
RsHKU3-7
SCoVrC
RsHKU3-12
RsHKU3-1
RaLYRa11
SW1
RstYN03
RmYN07
RstYN09
RpF46
SW
RspSC2018
SARS-CoV
SW2
RfYNLF/31C
RsAnlong103
RaYN2018A
Rpe3
Rf4092
As6526
RaYN2018C
Rs4081
Rs4255
Rs4247
Rs4237
Rs4231
Rs4874
Rs7327
Rs9401
Rs3367
RsSHC014
Rs4084
RaYN2018D
RaYN2018B

Figure S4: **Recombination break-points counted in genomic intervals of 1000 nt and deduced from the 5'- and 3'-ends of GRPSs characterized for the 11 viruses of the SCoV2rC *sensu lato* lineage.**

The genomic regions containing robust phylogenetic signal (GRPSs) were characterized using the SWB analysis based on a window of 2000 nt (see main text and **Figure 1** for more details).

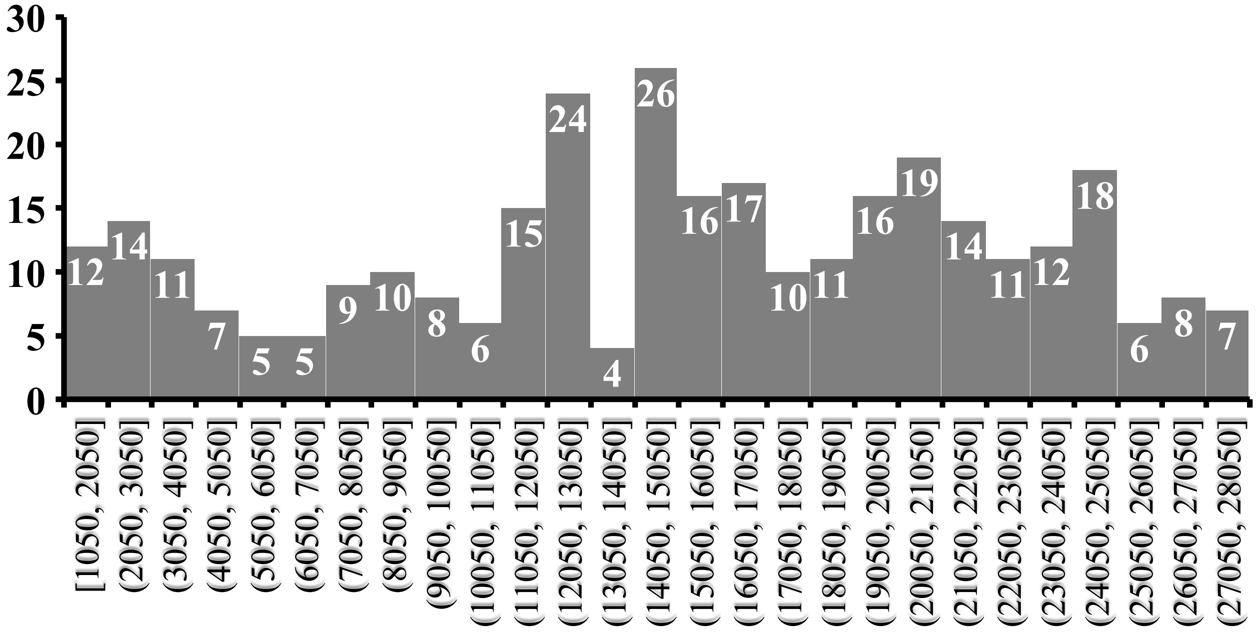

Table S1: Origin of the *Sarbecovirus* genomes used in this study

Virus name	Accession number	Host species	Geographic origin	Reference
SARS-CoV	NC_004718[1]	*Homo sapiens*	Canada	He et al. (2004)
As6526	KY417142[1]	*Aselliscus stoliczkanus*	Yunnan (China)	Hu et al. (2017)
RaLYRa11*	KF569996[1]	*Rhinolophus affinis*	Yunnan (China)	He et al. (2014)
RaYN2018A*	MK211375[1]	*Rhinolophus affinis*	Yunnan (China)	Han et al. (2019)
RaYN2018B*	MK211376[1]	*Rhinolophus affinis*	Yunnan (China)	Han et al. (2019)
RaYN2018C*	MK211377[1]	*Rhinolophus affinis*	Yunnan (China)	Han et al. (2019)
RaYN2018D*	MK211378[1]	*Rhinolophus affinis*	Yunnan (China)	Han et al. (2019)
Rf1	DQ412042[1]	*Rhinolophus ferrumequinum*[T1]	Hubei (China)	Li et al. (2005)
Rf4092	KY417145[1]	*Rhinolophus ferrumequinum*[T1]	Yunnan (China)	Hu et al. (2017)
RfJiyuan-84*	KY770860[1]	*Rhinolophus ferrumequinum*[T1]	Henan (China)	Lin et al. (2017)
RfV273*	DQ648856[1]	*Rhinolophus ferrumequinum*[T1]	Yunnan (China)	Tang et al. (2006)
RfYNLF/31C*	KP886808[1]	*Rhinolophus ferrumequinum*[T1]	Yunnan (China)	Lau et al. (2015)
RmYN07*	EPI_ISL_1699447[2]	*Rhinolophus malayanus*	Yunnan (China)	Zhou et al. (2021)
Rma1*	DQ412043[1]	*Rhinolophus macrotis*[T2]	Hubei (China)	Li et al. (2005)
RmaV279*	DQ648857[1]	*Rhinolophus macrotis*[T2]	Yunnan (China)	Tang et al. (2006)
RmoLongquan140*	KF294457[1]	*Rhinolophus monoceros*[T3]	Zhejiang (China)	Lin et al. (2017)
RpF46*	KU973692[1]	*Rhinolophus pusillus*	Yunnan (China)	Wang et al. (2017)
RpShaanxi2011	JX993987[1]	*Rhinolophus pusillus*	Shaanxi (China)	Yang et al. (2013)
Rpe3	DQ071615[1]	*Rhinolophus pearsoni*	Guangxi (China)	Li et al. (2005)
Rs3367	KC881006[1]	*Rhinolophus sinicus*	Yunnan (China)	Ge et al. (2013)
Rs4081	KY417143[1]	*Rhinolophus sinicus*	Yunnan (China)	Hu et al. (2017)
Rs4084	KY417144[1]	*Rhinolophus sinicus*	Yunnan (China)	Hu et al. (2017)
Rs4231	KY417146[1]	*Rhinolophus sinicus*	Yunnan (China)	Hu et al. (2017)
Rs4237	KY417147[1]	*Rhinolophus sinicus*	Yunnan (China)	Hu et al. (2017)
Rs4247	KY417148[1]	*Rhinolophus sinicus*	Yunnan (China)	Hu et al. (2017)
Rs4255	KY417149[1]	*Rhinolophus sinicus*	Yunnan (China)	Hu et al. (2017)
Rs4874	KY417150[1]	*Rhinolophus sinicus*	Yunnan (China)	Hu et al. (2017)
Rs7327	KY417151[1]	*Rhinolophus sinicus*	Yunnan (China)	Hu et al. (2017)
Rs9401	KY417152[1]	*Rhinolophus sinicus*	Yunnan (China)	Hu et al. (2017)
RsAnlong103*	KY770858[1]	*Rhinolophus sinicus*	Guizhou (China)	Lin et al. (2017)
RsHKU3-1*	DQ022305[1]	*Rhinolophus sinicus*	Hong-Kong (China)	Lau et al. (2005)
RsHKU3-7*	GQ153542[1]	*Rhinolophus sinicus*	Hong-Kong (China)	Lau et al. (2010)
RsHKU3-12*	GQ153547[1]	*Rhinolophus sinicus*	Hong-Kong (China)	Lau et al. (2010)
RsHuB2013*	KJ473814[1]	*Rhinolophus sinicus*	Hubei (China)	Wu et al. (2016)
RsSHC014	KC881005[1]	*Rhinolophus sinicus*	Yunnan (China)	Ge et al. (2013)
RstYN03*	EPI_ISL_1699443[2]	*Rhinolophus stheno*[T4]	Yunnan (China)	Zhou et al. (2021)
RstYN09*	EPI_ISL_1699449[2]	*Rhinolophus stheno*[T4]	Yunnan (China)	Zhou et al. (2021)
RspSC2018*	MK211374[1]	*Rhinolophus* sp.	Sichuan (China)	Han et al. (2019)
**SARS-CoV-2**	NC_045512[1]	*Homo sapiens*	Hubei (China)	Wu et al. (2020)
**RaTG13**	MN996532[1]	*Rhinolophus affinis*	Yunnan (China)	Zhou P. et al. (2020)

**RacCS203**	MW251308[1]	*Rhinolophus acuminatus*	Thailand	Wacharapluesadee et al. (2021)
**RmYN02***	EPI_ISL_412977[2]	*Rhinolophus malayanus*	Yunnan (China)	Zhou H. et al. (2020)
**RpYN06***	EPI_ISL_1699446[2]	*Rhinolophus pusillus*	Yunnan (China)	Zhou et al. (2021)
**RshSTT200**	EPI_ISL_852605[2]	*Rhinolophus shameli*	Cambodia	Delaune et al. (2021)
**MjGuangdong***	EPI_ISL_410721[2]	*Manis javanica*	Guangdong (China)	Xiao et al. (2020)
**MjGuangxi***	EPI_ISL_410539[2]	*Manis javanica*	Guangxi (China)	Lam et al. (2020)
**RsZXC21***	MG772934[1]	*Rhinolophus sinicus*	Zhejiang (China)	Hu et al. (2018)
**RsZC45***	MG772933[1]	*Rhinolophus sinicus*	Zhejiang (China)	Hu et al. (2018)
**RpPrC31***	EPI_ISL_1098866[2]	*Rhinolophus pusillus*	Yunnan (China)	Li et al. (2021)
**RaTG15**	GWHBAUP01000001[3]	*Rhinolophus affinis*	Yunnan (China)	Guo et al. (2021)
**Rst7931**	GWHBAUS01000001[3]	*Rhinolophus stheno*[T4]	Yunnan (China)	Guo et al. (2021)
**RmYN05**	EPI_ISL_1699445[2]	*Rhinolophus malayanus*	Yunnan (China)	Zhou et al. (2021)
**RstYN04***	EPI_ISL_1699444[2]	*Rhinolophus stheno*[T4]	Yunnan (China)	Zhou et al. (2021)
**Rc-o319**	LC556375[1]	*Rhinolophus cornutus*	Japan	Murakami et al. (2020)
**RbBM48-31***	NC_014470[1]	*Rhinolophus blasii*	Bulgaria	Drexler et al. (2010)
**RspKY72***	KY352407[1]	*Rhinolophus* sp.	Kenya	Tao and Tong (2019)

*original name slightly modified to be consistent with other names and to facilitate interpretations; 1: NCBI; 2: GISAID; 3: NGDC

Taxonomic issues (Burgin et al., 2020) = T1: currently *Rhinolophus nippon*; T2: currently *Rhinolophus episcopus*; T3 = the taxonomic assignation should be regarded as dubious because *Rhinolophus monoceros* is supposed to be endemic of Taiwan; T4: currently *Rhinolophus microglobosus*.

References

Drexler, J. F., Gloza-Rausch, F., Glende, J., Corman, V. M., Muth, D., Goettsche, M., Seebens, A., Niedrig, M., Pfefferle, S., Yordanov, S., Zhelyazkov, L., Hermanns, U., Vallo, P., Lukashev, A., Müller, M. A., Deng, H., Herrler, G., & Drosten, C. (2010). Genomic characterization of severe acute respiratory syndrome-related coronavirus in European bats and classification of coronaviruses based on partial RNA-dependent RNA polymerase gene sequences. Journal of virology, 84(21), 11336–11349. https://doi.org/10.1128/JVI.00650-10

Ge, X. Y., Li, J. L., Yang, X. L., Chmura, A. A., Zhu, G., Epstein, J. H., Mazet, J. K., Hu, B., Zhang, W., Peng, C., Zhang, Y. J., Luo, C. M., Tan, B., Wang, N., Zhu, Y., Crameri, G., Zhang, S. Y., Wang, L. F., Daszak, P., & Shi, Z. L. (2013). Isolation and characterization of a bat SARS-like coronavirus that uses the ACE2 receptor. Nature, 503(7477), 535–538. https://doi.org/10.1038/nature12711

Guo, H., Hu, B., Si, H. R., Zhu, Y., Zhang, W., Li, B., Li, A., Geng, R., Lin, H. F., Yang, X. L., Zhou, P., & Shi, Z. L. (2021). Identification of a novel lineage bat SARS-related coronaviruses that use bat ACE2 receptor. Emerging microbes & infections, 10(1), 1507–1514. https://doi.org/10.1080/22221751.2021.1956373

Han, Y., Du, J., Su, H., Zhang, J., Zhu, G., Zhang, S., Wu, Z., & Jin, Q. (2019). Identification of Diverse Bat Alphacoronaviruses and Betacoronaviruses in China Provides New Insights Into the Evolution and Origin of Coronavirus-Related Diseases. Frontiers in microbiology, 10, 1900. https://doi.org/10.3389/fmicb.2019.01900

He, B., Zhang, Y., Xu, L., Yang, W., Yang, F., Feng, Y., Xia, L., Zhou, J., Zhen, W., Feng, Y., Guo, H., Zhang, H., & Tu, C. (2014). Identification of diverse alphacoronaviruses and genomic characterization of a novel severe acute respiratory syndrome-like coronavirus from bats in China. Journal of virology, 88(12), 7070–7082. https://doi.org/10.1128/JVI.00631-14

He, R., Dobie, F., Ballantine, M., Leeson, A., Li, Y., Bastien, N., Cutts, T., Andonov, A., Cao, J., Booth, T. F., Plummer, F. A., Tyler, S., Baker, L., & Li, X. (2004). Analysis of multimerization of the SARS coronavirus nucleocapsid protein. Biochemical and biophysical research communications, 316(2), 476–483. https://doi.org/10.1016/j.bbrc.2004.02.074

Hu, B., Zeng, L. P., Yang, X. L., Ge, X. Y., Zhang, W., Li, B., Xie, J. Z., Shen, X. R., Zhang, Y. Z., Wang, N., Luo, D. S., Zheng, X. S., Wang, M. N., Daszak, P., Wang, L. F., Cui, J., & Shi, Z. L. (2017). Discovery of a rich gene pool of bat SARS-related coronaviruses provides new insights into the origin of SARS coronavirus. PLoS pathogens, 13(11), e1006698. https://doi.org/10.1371/journal.ppat.1006698

Hu, D., Zhu, C., Ai, L., He, T., Wang, Y., Ye, F., Yang, L., Ding, C., Zhu, X., Lv, R., Zhu, J., Hassan, B., Feng, Y., Tan, W., & Wang, C. (2018). Genomic characterization and infectivity of a novel SARS-like coronavirus in Chinese bats. Emerging microbes & infections, 7(1), 154. https://doi.org/10.1038/s41426-018-0155-5

Lam, T. T., Jia, N., Zhang, Y. W., Shum, M. H., Jiang, J. F., Zhu, H. C., Tong, Y. G., Shi, Y. X., Ni, X. B., Liao, Y. S., Li, W. J., Jiang, B. G., Wei, W., Yuan, T. T., Zheng, K., Cui, X. M., Li, J., Pei, G. Q., Qiang, X., Cheung, W. Y., … Cao, W. C. (2020). Identifying SARS-CoV-2-related coronaviruses in Malayan pangolins. Nature, 583(7815), 282–285. https://doi.org/10.1038/s41586-020-2169-0

Lau, S. K., Feng, Y., Chen, H., Luk, H. K., Yang, W. H., Li, K. S., Zhang, Y. Z., Huang, Y., Song, Z. Z., Chow, W. N., Fan, R. Y., Ahmed, S. S., Yeung, H. C., Lam, C. S., Cai, J. P., Wong, S. S., Chan, J. F., Yuen, K. Y., Zhang, H. L., & Woo, P. C. (2015). Severe Acute Respiratory Syndrome (SARS) Coronavirus ORF8 Protein Is Acquired from SARS-Related Coronavirus from Greater Horseshoe Bats through Recombination. Journal of virology, 89(20), 10532–10547. https://doi.org/10.1128/JVI.01048-15

Lau, S. K., Li, K. S., Huang, Y., Shek, C. T., Tse, H., Wang, M., Choi, G. K., Xu, H., Lam, C. S., Guo, R., Chan, K. H., Zheng, B. J., Woo, P. C., & Yuen, K. Y. (2010). Ecoepidemiology and complete genome comparison of different strains of severe acute respiratory syndrome-related Rhinolophus bat coronavirus in China reveal bats as a reservoir for acute, self-limiting infection that allows recombination events. Journal of virology, 84(6), 2808–2819. https://doi.org/10.1128/JVI.02219-09

Lau, S. K., Woo, P. C., Li, K. S., Huang, Y., Tsoi, H. W., Wong, B. H., Wong, S. S., Leung, S. Y., Chan, K. H., & Yuen, K. Y. (2005). Severe acute respiratory syndrome coronavirus-like virus in Chinese horseshoe bats. Proceedings of the National Academy of Sciences of the United States of America, 102(39), 14040–14045. https://doi.org/10.1073/pnas.0506735102

Li, W., Shi, Z., Yu, M., Ren, W., Smith, C., Epstein, J. H., Wang, H., Crameri, G., Hu, Z., Zhang, H., Zhang, J., McEachern, J., Field, H., Daszak, P., Eaton, B. T., Zhang, S., & Wang, L. F. (2005). Bats are natural reservoirs of SARS-like coronaviruses. Science (New York), 310(5748), 676–679. https://doi.org/10.1126/science.1118391

Lin, X. D., Wang, W., Hao, Z. Y., Wang, Z. X., Guo, W. P., Guan, X. Q., Wang, M. R., Wang, H. W., Zhou, R. H., Li, M. H., Tang, G. P., Wu, J., Holmes, E. C., & Zhang, Y. Z. (2017). Extensive diversity of coronaviruses in bats from China. Virology, 507, 1–10. https://doi.org/10.1016/j.virol.2017.03.019

Murakami, S., Kitamura, T., Suzuki, J., Sato, R., Aoi, T., Fujii, M., Matsugo, H., Kamiki, H., Ishida, H., Takenaka-Uema, A., Shimojima, M., & Horimoto, T. (2020). Detection and Characterization of Bat Sarbecovirus Phylogenetically Related to SARS-CoV-2, Japan. Emerging infectious diseases, 26(12), 3025–3029. https://doi.org/10.3201/eid2612.203386

Tang, X. C., Zhang, J. X., Zhang, S. Y., Wang, P., Fan, X. H., Li, L. F., Li, G., Dong, B. Q., Liu, W., Cheung, C. L., Xu, K. M., Song, W. J., Vijaykrishna, D., Poon, L. L., Peiris, J. S., Smith, G. J., Chen, H., & Guan, Y. (2006). Prevalence and genetic diversity of coronaviruses in bats from China. Journal of virology, 80(15), 7481–7490. https://doi.org/10.1128/JVI.00697-06

Tao, Y., & Tong, S. (2019). Complete Genome Sequence of a Severe Acute Respiratory Syndrome-Related Coronavirus from Kenyan Bats. Microbiology resource announcements, 8(28), e00548-19. https://doi.org/10.1128/MRA.00548-19

Wacharapluesadee, S., Tan, C. W., Maneeorn, P., Duengkae, P., Zhu, F., Joyjinda, Y., Kaewpom, T., Chia, W. N., Ampoot, W., Lim, B. L., Worachotsueptrakun, K., Chen, V. C., Sirichan, N., Ruchisrisarod, C., Rodpan, A., Noradechanon, K., Phaichana, T., Jantarat, N., Thongnumchaima, B., Tu, C., … Wang, L. F. (2021). Evidence for SARS-CoV-2 related coronaviruses circulating in bats and pangolins in Southeast Asia. Nature communications, 12(1), 972. https://doi.org/10.1038/s41467-021-21240-1

Wang, L., Fu, S., Cao, Y., Zhang, H., Feng, Y., Yang, W., Nie, K., Ma, X., & Liang, G. (2017). Discovery and genetic analysis of novel coronaviruses in least horseshoe bats in southwestern China. Emerging microbes & infections, 6(3), e14. https://doi.org/10.1038/emi.2016.140

Wu, F., Zhao, S., Yu, B., Chen, Y. M., Wang, W., Song, Z. G., Hu, Y., Tao, Z. W., Tian, J. H., Pei, Y. Y., Yuan, M. L., Zhang, Y. L., Dai, F. H., Liu, Y., Wang, Q. M., Zheng, J. J., Xu, L., Holmes, E. C., & Zhang, Y. Z. (2020). A new coronavirus associated with human respiratory disease in China. Nature, 579(7798), 265–269. https://doi.org/10.1038/s41586-020-2008-3

Wu, Z., Yang, L., Ren, X., Zhang, J., Yang, F., Zhang, S., & Jin, Q. (2016). ORF8-Related Genetic Evidence for Chinese Horseshoe Bats as the Source of Human Severe Acute Respiratory Syndrome Coronavirus. The Journal of infectious diseases, 213(4), 579–583. https://doi.org/10.1093/infdis/jiv476

Xiao, K., Zhai, J., Feng, Y., Zhou, N., Zhang, X., Zou, J. J., Li, N., Guo, Y., Li, X., Shen, X., Zhang, Z., Shu, F., Huang, W., Li, Y., Zhang, Z., Chen, R. A., Wu, Y. J., Peng, S. M., Huang, M., Xie, W. J., … Shen, Y. (2020). Isolation of SARS-CoV-2-related coronavirus from Malayan pangolins. Nature, 583(7815), 286–289. https://doi.org/10.1038/s41586-020-2313-x

Yang, L., Wu, Z., Ren, X., Yang, F., He, G., Zhang, J., Dong, J., Sun, L., Zhu, Y., Du, J., Zhang, S., & Jin, Q. (2013). Novel SARS-like betacoronaviruses in bats, China, 2011. Emerging infectious diseases, 19(6), 989–991. https://doi.org/10.3201/eid1906.121648

Zhou, H., Chen, X., Hu, T., Li, J., Song, H., Liu, Y., Wang, P., Liu, D., Yang, J., Holmes, E. C., Hughes, A. C., Bi, Y., & Shi, W. (2020). A Novel Bat Coronavirus Closely Related to SARS-CoV-2 Contains Natural Insertions at the S1/S2 Cleavage Site of the Spike Protein. Current biology : CB, 30(11), 2196–2203.e3. https://doi.org/10.1016/j.cub.2020.05.023

Zhou, H., Ji, J., Chen, X., Bi, Y., Li, J., Wang, Q., Hu, T., Song, H., Zhao, R., Chen, Y., Cui, M., Zhang, Y., Hughes, A. C., Holmes, E. C., & Shi, W. (2021). Identification of novel bat coronaviruses sheds light on the evolutionary origins of SARS-CoV-2 and related viruses. Cell, S0092-8674(21)00709-1. https://doi.org/10.1016/j.cell.2021.06.008

Zhou, P., Yang, X. L., Wang, X. G., Hu, B., Zhang, L., Zhang, W., Si, H. R., Zhu, Y., Li, B., Huang, C. L., Chen, H. D., Chen, J., Luo, Y., Guo, H., Jiang, R. D., Liu, M. Q., Chen, Y., Shen, X. R., Wang, X., Zheng, X. S., … Shi, Z. L. (2020). A pneumonia outbreak associated with a new coronavirus of probable bat origin. Nature, 579(7798), 270–273. https://doi.org/10.1038/s41586-020-2012-7
